# Carnosic acid enhances cisplatin sensitivity and suppresses gastric cancer progression via the TP53/SLC7A11/ALOX12 axis

**DOI:** 10.1186/s41065-025-00508-2

**Published:** 2025-07-23

**Authors:** Li Zhou, Bin Xu, Baojian Li

**Affiliations:** 1 Department of Traditional Chinese Medicine, Changzhou Fourth People’s Hospital, Changzhou City, Jiangsu Province 213032 China; 2 Department of Pharmacy, The Fourth People’s Hospital of Changzhou, No.68, Honghe Road, Xinbei District, Changzhou City, Jiangsu Province 213032 China

**Keywords:** Carnosic acid, Gastric cancer, Cisplatin, TP53

## Abstract

**Background:**

Gastric cancer (GC) remains a significant global health challenge due to its high mortality and frequent resistance to chemotherapy drugs like cisplatin (DDP). Carnosic acid (CA), a phenolic diterpene, exhibits potential anti-cancer properties. This study aimed to investigate the role of CA in regulating GC development and DDP sensitivity.

**Methods:**

The half-maximal inhibitory concentration (IC_50_) of DDP and cell viability were determined using a cell counting kit-8 assay. Cell proliferation was evaluated by a 5-Ethynyl-2’-deoxyuridine assay, while cell migration was assessed by a transwell assay. Cell death was analyzed through flow cytometry, fluorometric assay, and colorimetric assays. The targets of CA were identified using network pharmacology. Western blotting was employed to detect the protein expression of tumor protein p53 (TP53), solute carrier family 7 member 11 (SLC7A11), and arachidonate 12-lipoxygenase, 12 S type (ALOX12).

**Results:**

CA treatment significantly inhibited GC cell proliferation and migration and enhanced cell death. The treatment also elevated reactive oxygen species (ROS) and Fe^2+^ levels, while reducing glutathione (GSH) levels and the IC_50_ value for DDP in GC cells. In addition, TP53 was identified as a target of CA, and its protein expression was upregulated by CA treatment in GC cells. Silencing TP53 attenuated the effects of CA on cell proliferation, migration, death, and the sensitivity of tumor cells to DDP. Further, CA regulated the TP53-mediated SLC7A11/ALOX12 pathway.

**Conclusion:**

CA improved the sensitivity of GC cells to DDP and inhibited their malignant progression by regulating the TP53-mediated SLC7A11/ALOX12 axis, highlighting its potential clinical significance for GC treatment.

**Supplementary Information:**

The online version contains supplementary material available at 10.1186/s41065-025-00508-2.

## Highlights

CA treatment inhibited the malignant phenotypes of GC cells.

CA improved the sensitivity of GC cells to DDP.

TP53 was identified as a target of CA in GC cells.

TP53 knockdown relieved CA-induced effects in GC cells.

CA regulated TP53-mediated SLC7A11/ALOX12 pathway.

**Supplementary Information:**

The online version contains supplementary material available at 10.1186/s41065-025-00508-2.

## Introduction

Gastric cancer (GC) ranks as the fifth most common malignancy worldwide and is a leading cause of cancer-related mortality, with an estimated 968,350 new cases and 659,853 deaths annually [1]. *Helicobacter pylori* infection is a major risk factor for GC development [2]. Current non-surgical treatments primarily include radiotherapy and chemotherapy, which have limitations in terms of their efficacy and potential harm to normal tissues [3, 4]. For advanced GC, platinum-based chemotherapy regimens, particularly those incorporating cisplatin (DDP), remain a cornerstone of treatment [5, 6]. However, the landscape of cancer therapy is continuously evolving, moving beyond traditional cytotoxic agents towards more targeted and personalized strategies [7, 8]. Within this dynamic context, understanding how novel agents modulate treatment responses, such as enhancing DDP sensitivity, becomes crucial. This highlights the potential for repurposing or developing agents that can synergize with or overcome the limitations of established therapies, aligning with the broader shift towards innovative cancer treatment paradigms.

Carnosic acid (CA) is a natural polyphenolic diterpene compound primarily extracted from rosemary plants [9]. Due to the presence of phenolic groups in its molecular structure, CA is typically classified as a polyphenolic compound. This unique chemical composition endows CA with a variety of biological activities, including significant anti-inflammatory, antiviral, and antioxidant properties [10]. These characteristics position CA within the broader landscape of plant-derived anti-cancer agents, which have formed the basis of traditional medicine approaches for centuries. Notably, numerous phytochemicals from traditional Chinese medicine (TCM) systems demonstrate clinically relevant anti-tumor effects through diverse mechanisms. For example, Yanghe decoction inhibited colorectal cancer metastasis by modulating inflammatory pathways [11]. Guiqi Baizhu decoction enhanced the sensitivity of lung cancer to radiation via HIF-1α/DNA-PKcs axis suppression [12]. Triptonoterpene induced apoptosis in prostate cancer models [13]. Chemically, CA exhibits good solubility in organic solvents, structural stability, thermal resistance, and a favorable safety profile [9]. Previous studies also revealed its anti-tumor properties. For example, CA induced autophagy and activated Sestrin-2/LKB1/AMPK pathway to inhibit the malignant progression of lung cancer [14]. In addition, CA inhibited Akt and activated the AMPK pathway to inhibit prostate cancer cell proliferation [15]. Previous evidence has suggested its inhibitory effect on the malignant progression of GC cells [16]. CA has been shown to enhance sensitivity to DDP in models like oral squamous cell carcinoma [17]. However, the specific mechanisms by which CA inhibits GC progression and modulates DDP sensitivity remain unclear.

The TP53 tumor suppressor gene encodes the p53 protein, a critical nuclear transcription factor of cell division, DNA repair, and apoptosis [18]. Under various stress conditions, such as ultraviolet radiation, exposure to chemical toxins, hypoxic environments, excessive production of reactive oxygen species (ROS), and uncontrolled cell cycles, TP53 is activated, leading to stabilization of functional p53 protein [19]. Mutations in TP53 occur in over 50% of human cancers, and these mutations are under positive selection because they confer a significant advantage to cell survival and proliferation [20]. While TP53’s role in suppressing GC progression is established [21], its involvement in mediating CA’s effects in GC has not been investigated.

Based on this background, this study investigated the role of CA in GC progression and its underlying mechanisms. The current findings revealed that CA impeded GC cell malignancy by modulating the TP53 signaling pathway, offering novel insights for developing GC treatment strategies.

## Materials and methods

### Cell culture and treatment

SNU-1 (EK-Bioscience, Shanghai, China) and AGS (EK-Bioscience) were cultured in F12K medium (Yu Bo Biotech, Shanghai, China) and RPMI-1640 medium (WESTANG Biotech, Shanghai, China), respectively, at 37℃ with 5% CO_2_. In addition, to analyze the effects of CA on their biological behaviors, the cells were exposed to various doses of CA (1.527, 6.125, 12.25, 24, 25, 30, 50, 100, or 200 µg/mL, MedChemExpress, Princeton, NJ, USA) for 24 h.

### Cell transfection

The small interfering RNA of TP53 (si-TP53) and the matched control (si-NC) were provided by GenePharma (Shanghai, China). GC cells were digested and added to 12-well plates. Opti-MEM (Biocreative Technology, Beijing, China), siRNAs, and Lipofectamine 2000 (Thermo Fisher, Waltham, MA, USA) were mixed separately and added to each well. After 4–6 h of transfection, the medium was replaced with a fresh complete culture medium. After 24–48 h, the cell samples were used for the following assays.

### Cell counting kit-8 (CCK-8) assay

GC cells were digested with trypsin (BioDee BioTech, Beijing, China), centrifuged, and resuspended. The cell density was adjusted to 10^5^ cells/mL using complete medium, and 100 µL of the cell suspension was added to each well. After the cells had adhered, the medium was replaced with a fresh complete medium containing various concentrations of CA (MedChemExpress) or DDP (MedChemExpress). After 48 h, the 96-well plates were removed, and 10 µL of CCK-8 reagent (Beyotime, Shanghai, China) was added to each well. The cells were subjected to incubation in the dark for 1 h. The optical density (OD) was measured using a microplate reader, and cell viability was calculated. The half-maximal inhibitory concentration (IC_50_) of DDP or CA was analyzed based on 50% cell viability. The drug concentration and corresponding inhibition rate data were entered into the GraphPad Prism software. The “Nonlinear Regression” analysis method was selected, and the curve was automatically fitted and the IC_50_ value was calculated by the software. The assay was performed with three independent biological replicates.

### 5-Ethynyl-2’-deoxyuridine (EdU) assay

GC cells were seeded into 6-well plates and subjected to various treatments. The original medium was aspirated, and F12K medium (Yu Bo Biotech) or RPMI-1640 medium (WESTANG Biotech) was added to each well of the 6-well plates, followed by the addition of EdU working solution (20 µmol/L, Ribobio, Guangzhou, China). After labeling, 4% paraformaldehyde (Abace Biology, Beijing, China) was added to fix the cells, followed by permeabilization with 0.1% Triton X-100 (Think-Far Technology, Beijing, China) for 15 minutes. After washing twice with PBS, the cell nuclei were stained with 4’,6-Diamidino-2-Phenylindole (DAPI), and the cells were observed and photographed under a fluorescence microscope. The assay was performed with three independent biological replicates.

### Transwell assay

GC cells were seeded into 6-well plates and subjected to various treatments before being digested with trypsin and counted. The treated GC cells were resuspended in serum-free medium, and the cell suspension containing 3.5 × 10^4^ cells was added to the upper chambers of the migration assay. Normal nutrient medium supplemented with 20% fetal bovine serum was added to the lower chambers. The culture plates were then placed in the incubator for 24 h. The chambers were washed twice with PBS, and the migrated cells were fixed with 4% paraformaldehyde (Abace Biology) for 20 min and stained with crystal violet (Sigma, St. Louis, MO, USA). After removing the remaining cells in the upper chambers with a cotton swab, the migrated cells were observed and counted under a microscope. The assay was performed with three independent biological replicates.

### Flow cytometry analysis

GC cells to be tested were collected and washed with calcium- and magnesium-free PBS. The cells were centrifuged at 1000 rpm for 4 min to remove the PBS and then resuspended in a binding buffer solution. Fluorescent dyes used to analyze apoptotic and necrotic cells, Annexin V-FITC (Solarbio, Beijing, China) and PI (Solarbio), were added to the cells, which were then incubated for 15 min. An appropriate amount of buffer solution was added to the mix, followed by apoptotic and necrotic cell analysis using a flow cytometer. The assay was performed with three independent biological replicates.

### Analysis of reactive oxygen species (ROS) and glutathione (GSH)

ROS Fluorometric Assay Kit (Elabscience, Wuhan, China) and GSH Assay Kit (Abcam, Cambridge, MA, USA) were used to analyze ROS production and GSH levels in GC cells. The analysis of ROS and GSH levels was performed using a fluorescence microscope and microplate reader, respectively, according to the guidebooks. The assay was performed with three independent biological replicates.

### Fe^2+^ concentration analysis

Fe^2+^ levels were analyzed using the Fe^2+^ Microplate Assay Kit (absin, Shanghai, China). In brief, GC cells were harvested and sonicated, followed by exposure to Assay buffer. After centrifugation, the supernatant was collected and incubated with Reaction buffer and Dye Reagent for 1 h. The samples were analyzed using a microplate reader. The assay was performed with three independent biological replicates.

### Western blotting assay

After transfection, RIPA lysis buffer (Beyotime) was added to the culture dish for lysis on ice for 30 min. Protein samples were subjected to electrophoresis and then transferred to PVDF membranes at a constant current of 1.7 mA for 12 min. The PVDF membranes were incubated with the antibody against TP53 (DF7238, Affinity, Nanjing, China), cyclin-dependent kinase inhibitor 1 A (p21, AF6290, Affinity), mouse double minute 2 homolog (MDM2, AF0208, Affinity), SLC7A11 (DF12509, Affinity), ALOX12 (PA5-78760, Thermo Fisher), or β-actin (PA1-183, Thermo Fisher). The membranes were placed in an antibody incubation box containing the secondary antibody (Thermo Fisher) and incubated on a shaker for 90 min. The PVDF membranes were placed in a chemiluminescence imaging system for exposure and imaging. The assay was performed with three independent biological replicates.

### Prediction of CA targets and GC targets

The SwissTargetPrediction website (http://old.swisstargetprediction.ch/) was used to query the potential targets of “Carnosic Acid”. Subsequently, the genes related to gastric cancer were analyzed through the CTD database (https://ctdbase.org/). These targets and genes were uploaded to the Venny platform (https://bioinfogp.cnb.csic.es/tools/venny/index.html) to draw a Venn diagram and identify common targets.

### Protein-protein interaction (PPI) network

The intersection targets obtained through the aforementioned method were used to construct a PPI network in the STRING database (https://string-db.org/). The organism was set to “Homo sapiens”. The tsv file was imported into Cytoscape 3.9.1 software, and the “apply layout” feature was utilized to arrange the layout of the network graph, determining the positions of nodes and edges, while nodes not connected to the key network nodes were hidden. Potential therapeutic target genes were filtered based on their degree values.

### Kyoto encyclopedia of genes and genomes (KEGG) analysis

The SangerBox-biomedical data analysis toolbox (http://sangerbox.com/home.html) was utilized to perform KEGG analysis and generate diagrams for the CA targets in the treatment of GC.

### Molecular docking

To further analyze the binding ability of CA to the core targets, molecular docking simulations were performed. The SDF format of CA was downloaded from the PubChem database (https://pubchem.ncbi.nlm.nih.gov/). The PDB format structure of TP53 (PDB ID: 5E1S) was downloaded from the RCSB (https://www.rcsb.org/). Finally, molecular docking simulations were performed using Autodock 1.517 software, and the docking conformations were visualized using Pymol software.

### Statistical analysis

All statistical analyses and figure generation were performed using GraphPad Prism 8.0. All continuous variable data were expressed as mean ± standard deviation and were compared using one-way ANOVA. The significance level was set at *P* = 0.05, where *P* < 0.05 indicated a statistically significant difference.

## Results

### CA treatment inhibited GC cell proliferation and migration and promoted cell death and the sensitivity of tumor cells to DDP

**Fig. 1:**
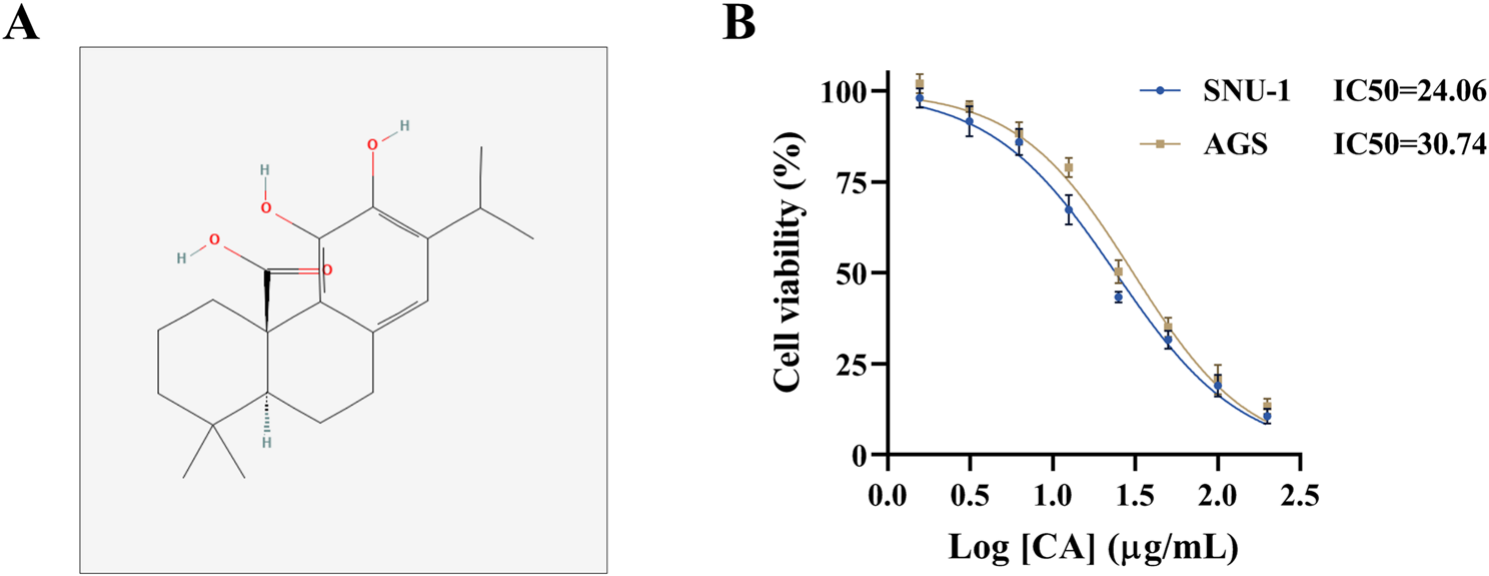
**Analysis of IC**_**50**_ values of DDP in SNU-1 and AGS cells. (**A**) The 2D structure diagram of the CA. (**B**) CCK-8 assay was performed to detect IC_50_ values of DDP in SNU-1 and AGS cells

**Fig. 2:**
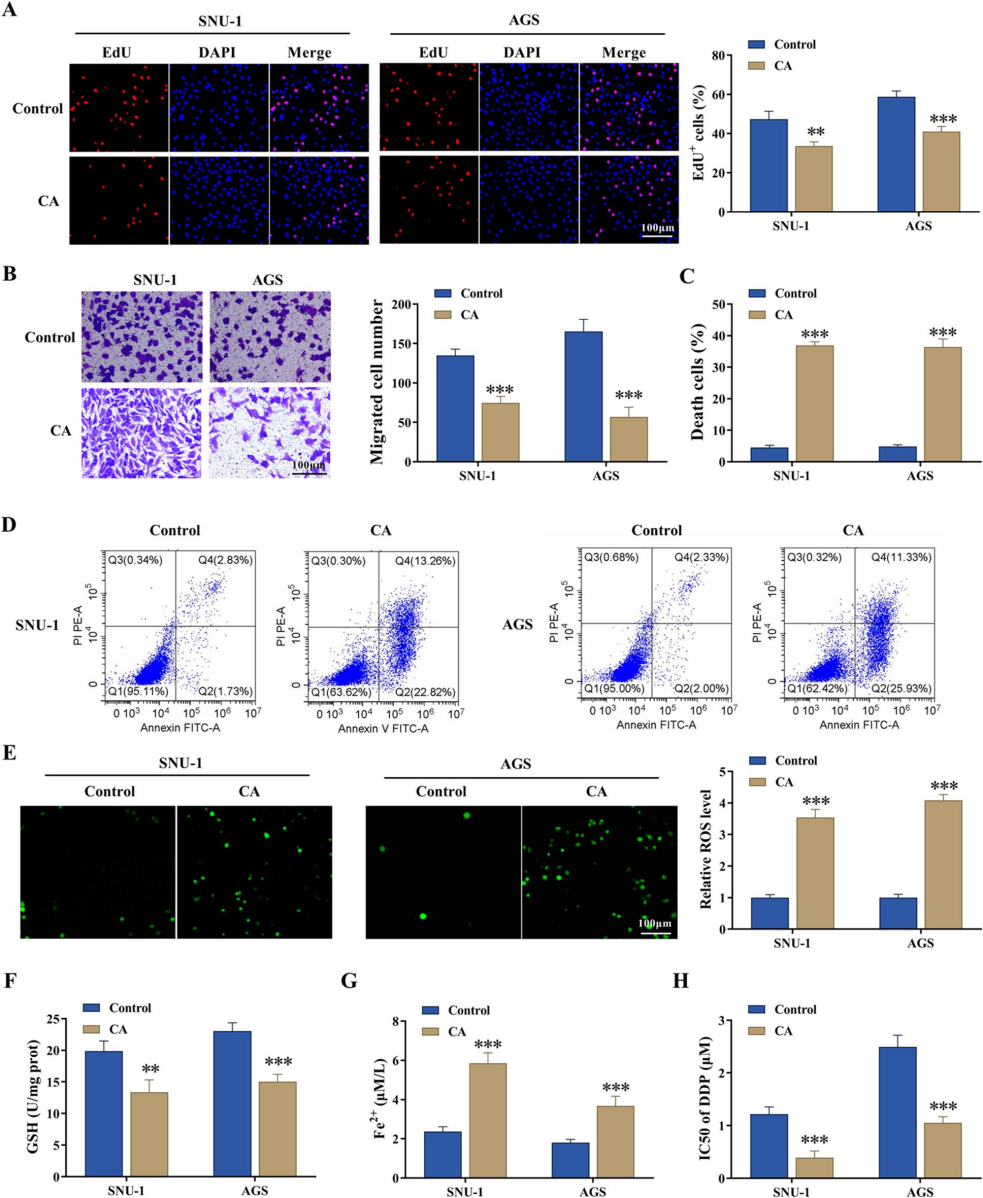
CA treatment inhibited GC cell proliferation and migration and promoted cell death and the sensitivity of tumor cells to DDP. SNU-1 and AGS cells were treated with CA (24 µg/mL and 30.74 µg/mL, respectively) or saline. (**A**) Cell proliferation was analyzed by EdU assay. (**B**) Cell migration was assessed by transwell assay. (**C** and **D**) Cell apoptosis and necrosis were analyzed by flow cytometry. (**E**) ROS levels were assessed by a fluorometric assay. (**F** and **G**) GSH and Fe^2+^ levels were detected by colorimetric assays. (H) CCK-8 assay was performed to detect IC_50_ values of DDP. **P < 0.01 and ***P < 0.001

The study analyzed the effect of CA on the malignant progression of SNU-1 and AGS cells and DDP sensitivity. The 2D structure diagram of the CA is shown in Fig. 1A. The IC_50_ values of DDP in SNU-1 and AGS cells were analyzed by CCK-8 assays. The results showed that their IC_50_ values were 24.06 and 30.74, respectively (Fig. 1B). Based on the result, the study treated SNU-1 and AGS cells with 24 µg/mL CA and 30 µg/mL CA, respectively. Subsequent data showed that CA treatment inhibited cell proliferation and migration and induced cell apoptosis and necrosis (Fig. 2A-D). The results also showed that CA treatment increased ROS and Fe^2+^ levels and decreased GSH levels (Fig. 2E-G). Moreover, the IC_50_ value of DDP was decreased after CA treatment in both SNU-1 and AGS cells (Fig. 2H). These data demonstrated that CA treatment improved DDP sensitivity and inhibited the malignant progression of GC cells.

### TP53 was identified as a target of CA through the network Pharmacology

**Fig. 3:**
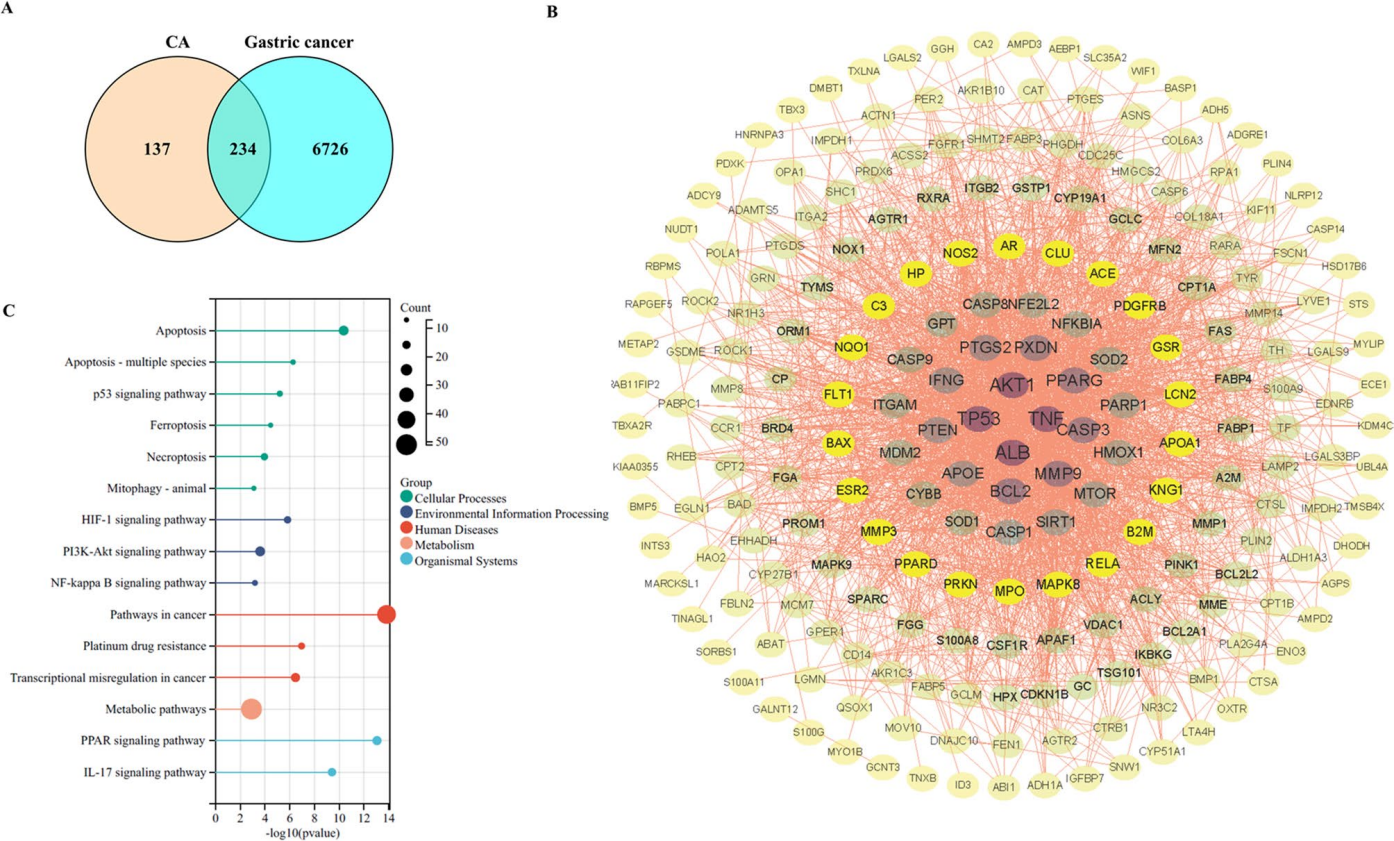
Analysis of common targets of CA and GC progression. (**A**) The study analyzed the CA targets and GC targets and then used a Venn diagram to identify the common targets. (**B**) PPI network of 234 genes was analyzed using the STRING database. (**C**) The KEGG pathway analysis of these 234 genes

**Fig. 4:**
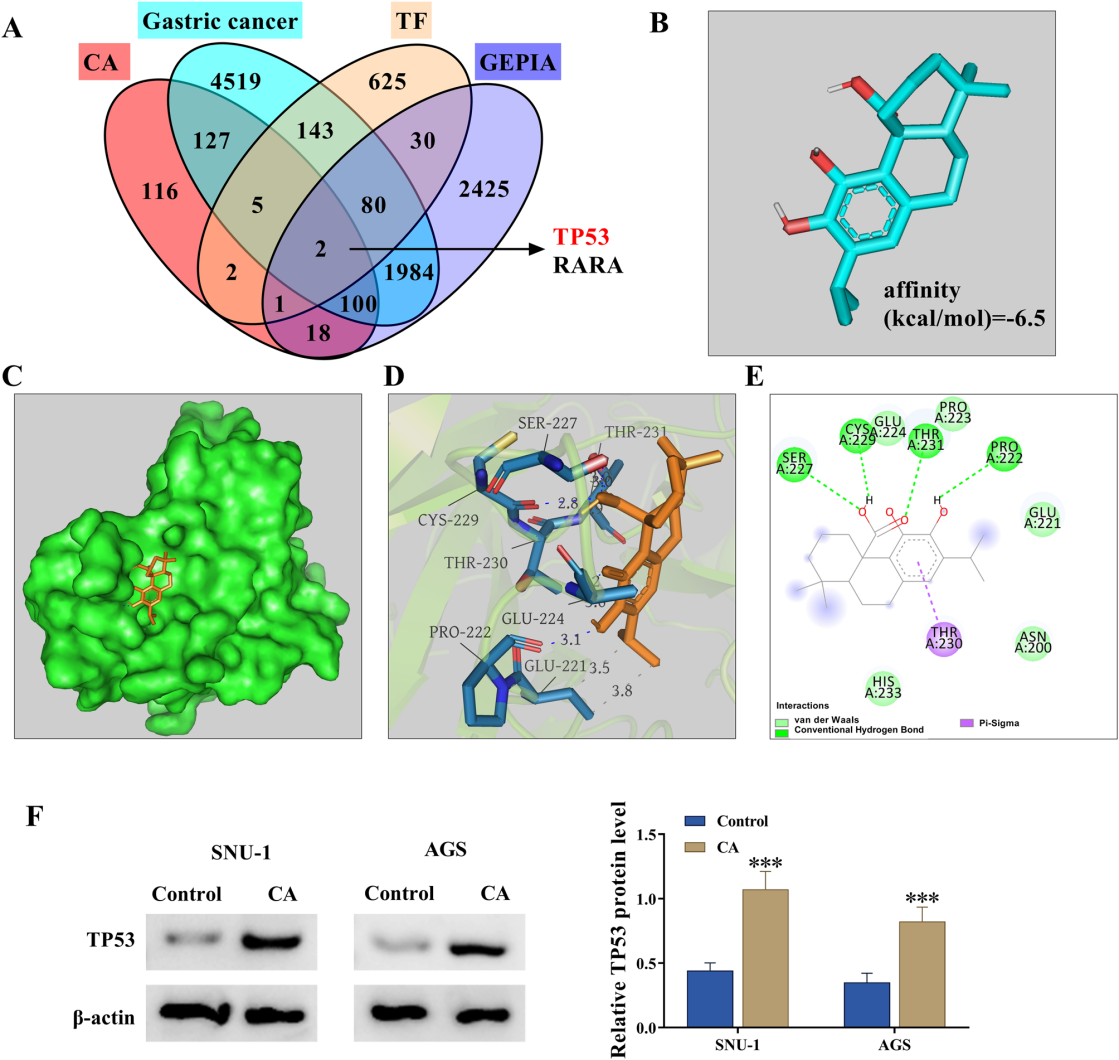
TP53 was identified as a target of CA. (**A**) The Venn diagram was used to identify the comment genes in CA targets, GC targets, TF, and GC tissues. (**B**) The conformation of TP53. (**C**-**D**) The 3D visualization of the biomolecular interaction between CA and the TP53 protein. (E) The 2D binding visualization between CA and the TP53 protein. (**F**) The effect of CA on the protein expression of TP53 was analyzed by western blotting assay in both SNU-1 and AGS cells. ***P < 0.001

The study also analyzed the CA targets and GC targets and then used a Venn diagram to identify the common targets. The result showed that there were 234 common targets (Fig. 3A). The protein-protein interaction (PPI) networks of 234 genes were analyzed using the STRING database, and the result is shown in Fig. 3B. The top four genes, ranked by degree values, were AKT1, TP53, ALB and TNF. The KEGG analysis showed that 234 genes were associated with apoptosis, ferroptosis, and platinum drug resistance (Fig. 3C). Subsequently, the Venn diagram was used to identify the comment genes in CA targets, GC targets, transcriptional factor (TF), and GC tissues. As shown in Fig. 4A, the common genes included TP53 and RARA. Based on the above results, the study employed TP53 as a common target of CA. The conformation of TP53 is shown in Fig. 4B. The PyMOL software was used for the 3D visualization of the biomolecular interaction between CA and the TP53 protein, and the result is shown in Fig. 4C and D (binding energy − 6.5 kcal/mol). The 2D binding relationship between them is shown in Fig. 4E. Further data showed that CA treatment promoted the protein expression of TP53, p21 and MDM2 in SNU-1 and AGS cells (Fig. 4F and Fig S1A and B). Thus, TP53 was a target of CA in GC cells.

### TP53 Silencing attenuated CA-induced effects in SNU-1 and AGS cells

**Fig. 5:**
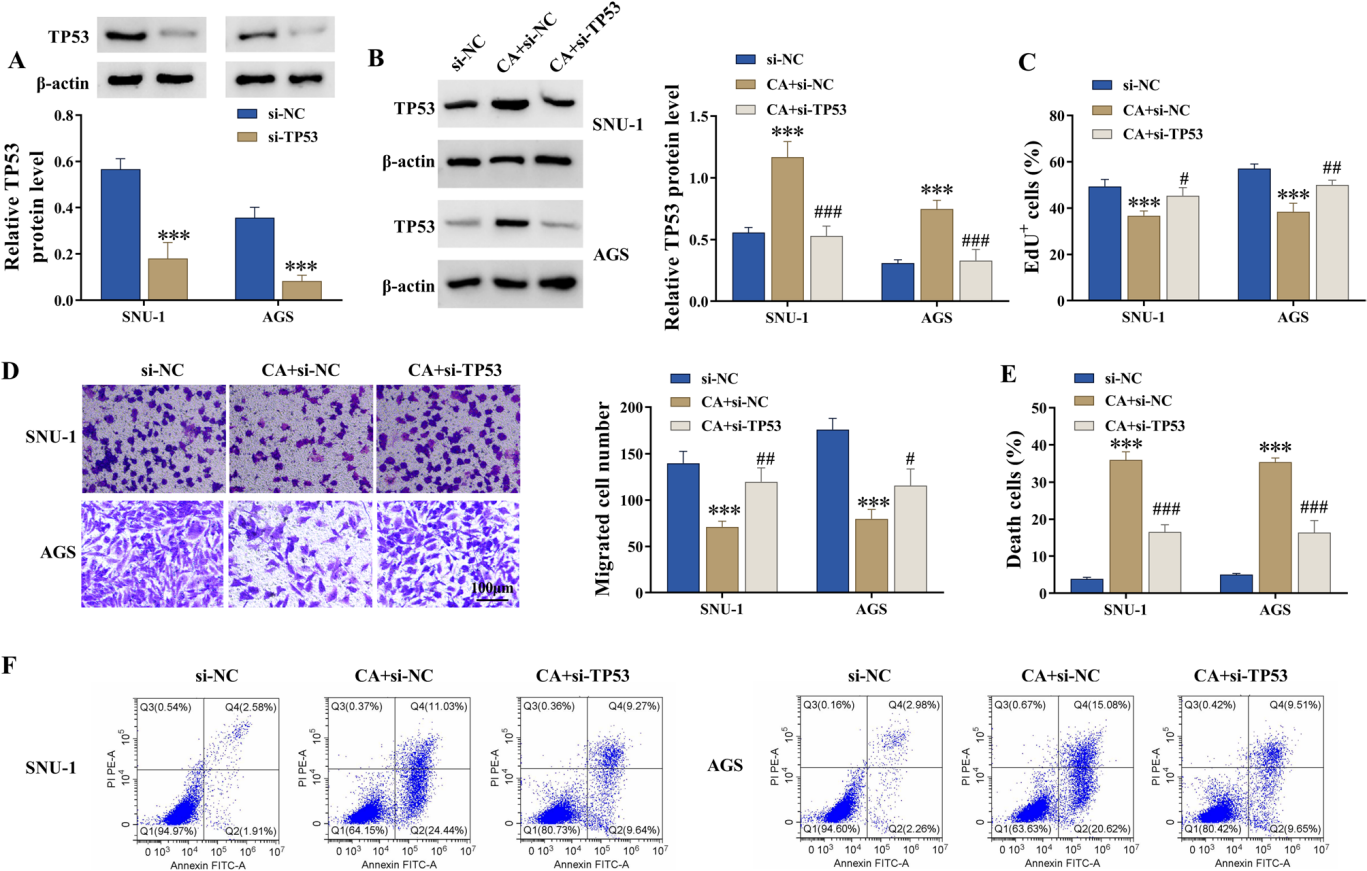
CA inhibited the malignant progression of GC cells by regulating TP53. (**A**) The efficiency of TP53 knockdown was analyzed by western blotting assay. (B-F) SNU-1 and AGS cells were divided into three groups, including si-NC group, CA + si-NC group, and CA + si-TP53 group. (**B**) TP53 protein expression was detected by western blotting assay. (**C**) Cell proliferation was analyzed by EdU assay. (**D**) Cell migration was assessed by transwell assay. (E and F) Cell apoptosis and necrosis were analyzed by flow cytometry. ***P < 0.001, # *P* < 0.05, ## *P* < 0.01, and ### *P* < 0.001

**Fig. 6:**
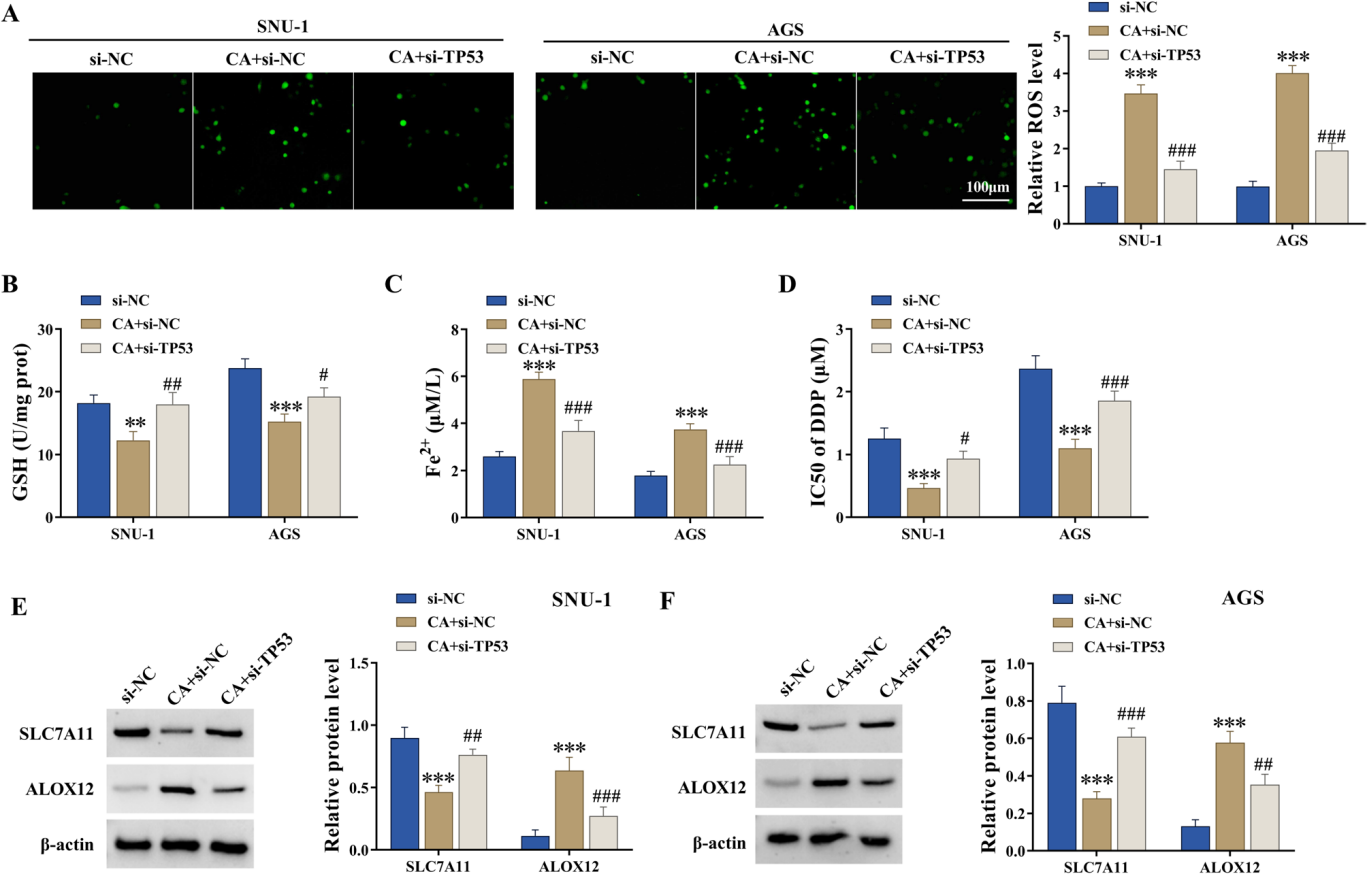
CA enhanced DDP sensitivity and regulated the SLC7A11/ALOX12 axis through TP53. (A-F) SNU-1 and AGS cells were divided into three groups, including si-NC group, CA + si-NC group, and CA + si-TP53 group. (**A**) ROS levels were assessed by the fluorometric assay. (**B** and **C**) GSH and Fe^2+^ levels were detected by colorimetric assays. (**D**) CCK-8 assay was performed to detect IC50 values of DDP. (**E** and **F**) The protein expression of SLC7A11 and ALOX12 was detected by western blotting assay. ***P < 0.001, # *P* < 0.05, ## *P* < 0.01, and ### *P* < 0.001

The study then analyzed the association of CA and TP53 in regulating the malignant progression of GC cells and DDP sensitivity. To achieve this, the study transfected SNU-1 and AGS cells with TP53 siRNA, followed by CA treatment. The efficiency of TP53 knockdown is shown in Fig. 5A. Subsequently, the result showed that CA treatment promoted TP53 protein expression, whereas the effect was attenuated after transfection with TP53 siRNA (Fig. 5B). The results also showed that CA treatment inhibited cell proliferation and migration and induced cell apoptosis and necrosis, whereas these effects were relieved after TP53 knockdown (Fig. 5C-F). In addition, TP53 silencing attenuated the promoting effects of CA on ROS and Fe^2+^ levels and its inhibitory effect on GSH levels (Fig. 6A-C). The CA treatment-induced inhibitory effect on the IC_50_ value of DDP was restored after TP53 silencing (Fig. 6D). The study further analyzed whether CA regulated the TP53-mediated SLC7A11/ALOX12 pathway in SNU-1 and AGS cells. To end this, the study transfected TP53 siRNA or si-NC into SNU-1 and AGS cells, followed by CA treatment. As shown in Fig. 6E and F, CA treatment inhibited SLC7A11 protein expression and promoted ALOX12 protein expression, however, these effects were attenuated after transfection with TP53 siRNA. Thus, CA inhibited the malignant progression of GC cells and improved DDP sensitivity by regulating the TP53**/**SLC7A11/ALOX12 axis.

## Discussion

Gastric cancer, as a common malignant tumor, poses a serious threat to human health, with Helicobacter pylori infection being a major etiological factor linked to disease progression and metastasis [22, 23]. In the treatment of advanced GC, chemotherapy plays a crucial role. Among the chemotherapeutic agents, DDP is widely used [24], however, the issue of chemotherapy resistance to DDP remains a significant challenge in clinical treatment [25]. Notably, emerging evidence suggests H. pylori infection may influence therapeutic responses and tumor aggressiveness [26], underscoring the need to contextualize resistance mechanisms within GC’s multifactorial pathogenesis. CA, a phenolic diterpene compound with anti-cancer activity, has not been thoroughly investigated in terms of its regulatory mechanism in GC and its role in enhancing the sensitivity of GC cells to cisplatin. This study aimed to address this gap. The current results demonstrated that CA promoted TP53 expression to decrease SLC7A11 and increase ALOX12 expression, thus enhancing DDP sensitivity and inhibiting the malignant progression of GC cells. This CA-mediated pathway may offer a strategy to counteract resistance mechanisms potentially exacerbated by factors like H. pylori infection.

The present study adds significant depth to the existing body of research on the anticancer properties of CA. While previous studies highlighted CA’s ability to induce apoptosis in breast and colorectal cancer via albumin nanoparticles [27], and trigger apoptosis while inhibiting Akt/mTOR signaling in GC [16], the precise mechanisms underlying its impact on GC chemosensitivity were unclear. The current study addressed this gap by demonstrating that CA treatment significantly inhibited GC cell proliferation and migration, while enhancing cell apoptosis, necrosis, and ferroptosis. A novel finding of this study was the synergistic effect of CA on the sensitivity of GC cells to DDP, positioning it as a promising chemoadjuvant.

Mechanistically, the study identified TP53 pathway as a critical mediator of CA’s effects in GC. TP53’s role in GC pathogenesis and treatment response is well-documented, influencing chemotherapy and immunotherapy outcomes through mutations and loss of heterozygosity [28]. It has been implicated in inducing ferroptosis via SLC7A11 downregulation [29], and suppressing metastasis via pathways like EEF1AKMT3/MAP2K7/TP53 [21]. The current data solidified this connection: CA treatment robustly increased TP53, p21 and MDM2 expression, and TP53 knockdown markedly attenuated CA’s inhibitory effects. Furthermore, the study delineated a downstream pathway where CA, via TP53, suppresses SLC7A11 expression and enhances ALOX12 expression. As a transcription factor, TP53 can directly bind to the promoter region of the SLC7A11 gene, inhibit its transcription, and thereby reduce the expression of the SLC7A11 protein. However, this still requires experimental verification. Biologically, SLC7A11 knockdown promotes ALOX12’s lipoxygenase activity to increase the peroxidation of PUFA-containing phospholipids, ultimately inducing ferroptosis and thereby inhibiting tumor progression [30]. Notably, while SLC7A11 downregulation and ALOX12 upregulation were consistently observed in SNU-1 and AGS cells, the study noted cell context-dependent variations in the magnitude of SLC7A11 and ALOX12 induction. This suggests potential modulation by tumor-specific factors such as epigenetic states or co-regulators, warranting further investigation into auxiliary mechanisms fine-tuning SLC7A11 and ALOX12 activity. It is also essential to situate these findings within the broader landscape of cancer drug resistance. Resistance mechanisms often involve complex, conserved pathways across tumor types. For instance, resistance in hepatocellular carcinoma involves the p-MYH9/USP22 axis stabilizing HIF-1alpha, promoting stemness and lenvatinib resistance, while in pancreatic cancer, targeting MXD1 overcomes trametinib resistance [31]. The current identification of the TP53/SLC7A11/ALOX12 axis as a modulator of DDP sensitivity adds a novel ferroptosis-linked pathway to this compendium of resistance mechanisms, highlighting potential convergent therapeutic strategies targeting key regulatory nodes across different cancers.

However, several limitations warrant discussion. First, all findings derive from in vitro models, lacking validation in patient-derived tissues, animal models, or clinically relevant systems. This restricts direct translation to human pathophysiology and precludes assessment of CA’s effects on tumor-microenvironment crosstalk or systemic toxicity. Future studies should prioritize in vivo validation using patient-derived xenograft (PDX) models, which preserve tumor heterogeneity, stromal interactions, and drug response profiles seen in patients [32, 33]. In addition, pharmacokinetic properties of CA (e.g., bioavailability, tissue distribution, metabolism) remain uncharacterized in vivo. Effective delivery to gastric tumors may require advanced strategies, such as nanoparticle encapsulation. Exploring these approaches will be essential to translate CA’s in vitro potency into feasible therapies. Furthermore, the clinical heterogeneity of GC demands consideration. While the current study utilized common GC cell lines, exploring CA’s efficacy and this mechanism in rarer, aggressive subtypes like hepatoid adenocarcinoma of the stomach (HAS) is crucial. HAS exhibits distinct clinicopathological features and often carries a poor prognosis [34]. Understanding whether the TP53/SLC7A11/ALOX12 axis is functional and targetable in such resistant subtypes could significantly impact treatment strategies for these challenging GC variants.

In conclusion, this study advanced the field by providing new insights into the molecular mechanisms by which CA inhibited GC cell growth and enhanced chemotherapy sensitivity. The identification of TP53 as a key mediator of CA’s effects opens new avenues for research into targeted therapies for GC.

## Electronic supplementary material

Below is the link to the electronic supplementary material.

Supplementary Material 1:Figure S1 The effect of CA on the protein expression of p21 and MDM2 was analyzed by western blotting assay in both SNU-1 (A) and AGS cells (B). **P 003C 0.01 and ***P 003C 0.001.

## Data Availability

No datasets were generated or analysed during the current study.

## References

[1] Bray F, Laversanne M, Sung H, Ferlay J, Siegel RL, Soerjomataram I, Jemal A. Global cancer statistics 2022: GLOBOCAN estimates of incidence and mortality worldwide for 36 cancers in 185 countries. CA Cancer J Clin. 2024;74(3):229–63. 38572751 10.3322/caac.21834

[2] Alipour M. Molecular mechanism of Helicobacter pylori-Induced gastric Cancer. J Gastrointest Cancer. 2021;52(1):23–30. 32926335 10.1007/s12029-020-00518-5 PMC7487264

[3] Guan WL, He Y, Xu RH. Gastric cancer treatment: recent progress and future perspectives. J Hematol Oncol. 2023;16(1):57. 37245017 10.1186/s13045-023-01451-3 PMC10225110

[4] Maryam A, Zahra S, Soghra F, Zeynab M. Radioprotective potency of nanoceria. Curr Radiopharmaceuticals. 2024;17(2):138–47. 10.2174/0118744710267281231104170435 37990425

[5] Badie A, Gaiddon C, Mellitzer G. Histone deacetylase functions in gastric Cancer. Therapeutic Target? Cancers 2022;14(21):5472. 10.3390/cancers14215472 PMC9659209 36358890

[6] Liu C, Li S, Tang Y. Mechanism of cisplatin resistance in gastric cancer and associated MicroRNAs. Cancer Chemother Pharmacol. 2023;92(5):329–40. 37535106 10.1007/s00280-023-04572-1

[7] Sonkin D, Thomas A, Teicher BA. Cancer treatments: past, present, and future. Cancer Genet. 2024;286–287:18–24. 38909530 10.1016/j.cancergen.2024.06.002 PMC11338712

[8] Liu H, Dilger JP. Different strategies for cancer treatment: targeting cancer cells or their neighbors? Chin J Cancer Res. 2025;37(2):289–92. 40353083 10.21147/j.issn.1000-9604.2025.02.12 PMC12062981

[9] Chen X, Wei C, Zhao J, Zhou D, Wang Y, Zhang S, Zuo H, Dong J, Zhao Z, Hao M, et al. Carnosic acid: an effective phenolic diterpenoid for prevention and management of cancers via targeting multiple signaling pathways. Pharmacol Res. 2024;206:107288. 38977208 10.1016/j.phrs.2024.107288

[10] Mirza FJ, Zahid S, Holsinger RMD. Neuroprotective effects of carnosic acid: insight into its mechanisms of action. Molecules 2023;28(5):2306. 10.3390/molecules28052306 PMC10005014 36903551

[11] Zhang L, Liu S, Ding K, Zeng B, Li B, Zhou J, Li J, Wang J, Su X, Sun R. Yanghe Decoction inhibits inflammation-induced lung metastasis of colorectal cancer. J Ethnopharmacol. 2025;340:119257. 39694428 10.1016/j.jep.2024.119257

[12] Li Y, Yang G, Li Q, Zhang Y, Zhang S, Zhou T, Wang X, Liu F, Miao Z, Qi Y, et al. Guiqi Baizhu Decoction enhances radiosensitivity in non-small cell lung cancer by inhibiting the HIF-1α/DNA-PKcs axis-mediated DNA repair. Phytomedicine: Int J Phytotherapy Phytopharmacology. 2025;140:156591. 10.1016/j.phymed.2025.156591 40054178

[13] Sang Y, Hu Y, Zhang Y, Chen L, Lu Y, Gao L, Lu Y, Cao X, Zhang Y, Chen G. Network pharmacology, molecular Docking and biological verification to explore the potential anti-prostate cancer mechanisms of tripterygium wilfordii hook. F. J Ethnopharmacol. 2025;338(Pt 2):119071. 39522845 10.1016/j.jep.2024.119071

[14] O’Neill EJ, Sze NSK, MacPherson REK, Tsiani E. Carnosic acid against lung cancer: induction of autophagy and activation of Sestrin-2/LKB1/AMPK signalling. Int J Mol Sci 2024;25(4):1950. 10.3390/ijms25041950 PMC10888478 38396629

[15] Nadile M, Sze NSK, Fajardo VA, Tsiani E. Inhibition of prostate Cancer cell survival and proliferation by carnosic acid is associated with Inhibition of Akt and activation of AMPK signaling. Nutrients 2024;16(9):1257. 10.3390/nu16091257 PMC11085396 38732504

[16] El-Huneidi W, Bajbouj K, Muhammad JS, Vinod A, Shafarin J, Khoder G, Saleh MA, Taneera J, Abu-Gharbieh E. Carnosic acid induces apoptosis and inhibits akt/mtor signaling in human gastric Cancer cell lines. Pharmaceuticals (Basel) 2021;14(3):230. 10.3390/ph14030230 PMC7998299 33800129

[17] Han L, Li L, Wu G. Induction of ferroptosis by carnosic acid-mediated inactivation of Nrf2/HO-1 potentiates cisplatin responsiveness in OSCC cells. Mol Cell Probes. 2022;64:101821. 35490795 10.1016/j.mcp.2022.101821

[18] Hassin O, Oren M. Drugging p53 in cancer: one protein, many targets. Nat Rev Drug Discovery. 2023;22(2):127–44. 36216888 10.1038/s41573-022-00571-8 PMC9549847

[19] Capuozzo M, Santorsola M, Bocchetti M, Perri F, Cascella M, Granata V, Celotto V, Gualillo O, Cossu AM, Nasti G et al.: p53: from fundamental biology to clinical applications in Cancer. Biology 2022;11(9):1325. 10.3390/biology11091325 PMC9495382 36138802

[20] Voskarides K, Giannopoulou N. The role of TP53 in adaptation and evolution. Cells 2023;12(3):512. 10.3390/cells12030512 PMC9914165 36766853

[21] Hong YH, Aziz N, Park JG, Lee D, Kim JK, Kim SA, Choi W, Lee CY, Lee HP, Huyen Trang HT, et al. The EEF1AKMT3/MAP2K7/TP53 axis suppresses tumor invasiveness and metastasis in gastric cancer. Cancer Lett. 2022;544:215803. 35753528 10.1016/j.canlet.2022.215803

[22] Salvatori S, Marafini I, Laudisi F, Monteleone G, Stolfi C. Helicobacter pylori and gastric cancer: pathogenetic mechanisms. Int J Mol Sci 2023;24(3):2895. 10.3390/ijms24032895 PMC9917787 36769214

[23] Viana D, Fatemeh Z, Simin N, Fatemeh N. An immunoinformatic approach to designing a Multi-epitope vaccine against < i > helicobacter pylori with the VacA toxin and BabA adhesion. Curr Proteomics. 2024;21(2):97–112.

[24] Yuan L, Xu ZY, Ruan SM, Mo S, Qin JJ, Cheng XD. Long non-coding RNAs towards precision medicine in gastric cancer: early diagnosis, treatment, and drug resistance. Mol Cancer. 2020;19(1):96. 32460771 10.1186/s12943-020-01219-0 PMC7251695

[25] Wang X, Xu Z, Sun J, Lv H, Wang Y, Ni Y, Chen S, Hu C, Wang L, Chen W, et al. Cisplatin resistance in gastric cancer cells is involved with GPR30-mediated epithelial-mesenchymal transition. J Cell Mol Med. 2020;24(6):3625–33. 32052561 10.1111/jcmm.15055 PMC7131920

[26] Ou L, Liu H, Peng C, Zou Y, Jia J, Li H, Feng Z, Zhang G, Yao M. Helicobacter pylori infection facilitates cell migration and potentially impact clinical outcomes in gastric cancer. Heliyon. 2024;10(17):e37046. 39286209 10.1016/j.heliyon.2024.e37046 PMC11402937

[27] Khella KF, Abd El Maksoud AI, Hassan A, Abdel-Ghany SE, Elsanhoty RM, Aladhadh MA, Abdel-Hakeem MA. Carnosic acid encapsulated in albumin nanoparticles induces apoptosis in breast and colorectal Cancer cells. Molecules 2022;27(13):4102. 10.3390/molecules27134102 PMC9268188 35807348

[28] Gu Y, Sun M, Fang H, Shao F, Lin C, Liu H, Li H, He H, Li R, Wang J, et al. Impact of clonal TP53 mutations with loss of heterozygosity on adjuvant chemotherapy and immunotherapy in gastric cancer. Br J Cancer. 2024;131(8):1320–7. 39217196 10.1038/s41416-024-02825-1 PMC11473753

[29] Guan Z, Chen J, Li X, Dong N. Tanshinone IIA induces ferroptosis in gastric cancer cells through p53-mediated SLC7A11 down-regulation. Biosci Rep 2020;40(8):BSR20201807. 10.1042/BSR20201807 PMC7953492 32776119

[30] Chu B, Kon N, Chen D, Li T, Liu T, Jiang L, Song S, Tavana O, Gu W. ALOX12 is required for p53-mediated tumour suppression through a distinct ferroptosis pathway. Nat Cell Biol. 2019;21(5):579–91. 30962574 10.1038/s41556-019-0305-6 PMC6624840

[31] Zhang S, Deng S, Liu J, Liu S, Chen Z, Liu S, Xue C, Zeng L, Zhao H, Xu Z et al. Targeting MXD1 sensitises pancreatic cancer to Trametinib. Gut 2025;74(8):1262-78. 10.1136/gutjnl-2024-333408 39819860

[32] Abdolahi S, Ghazvinian Z, Muhammadnejad S, Saleh M, Asadzadeh Aghdaei H, Baghaei K. Patient-derived xenograft (PDX) models, applications and challenges in cancer research. J Transl Med. 2022;20(1):206. 35538576 10.1186/s12967-022-03405-8 PMC9088152

[33] Liu Y, Wu W, Cai C, Zhang H, Shen H, Han Y. Patient-derived xenograft models in cancer therapy: technologies and applications. Signal Transduct Target Ther. 2023;8(1):160. 37045827 10.1038/s41392-023-01419-2 PMC10097874

[34] Xia R, Zhou Y, Wang Y, Yuan J, Ma X. Hepatoid adenocarcinoma of the stomach: current perspectives and new developments. Front Oncol. 2021;11:633916. 33912455 10.3389/fonc.2021.633916 PMC8071951

