# Supplementary material for: Carnosic acid enhances cisplatin sensitivity and suppresses gastric cancer progression via the TP53/SLC7A11/ALOX12 axis
Source: Hereditas. 2025 Jul 23;162:139. doi: 10.1186/s41065-025-00508-2 (PMC12285127; doi:10.1186/s41065-025-00508-2)
Supplement: Supplementary file 1 — Supplementary Material 1:Figure S1 The effect of CA on the protein expression of p21 and MDM2 was analyzed by western blotting assay in both SNU-1 (A) and AGS cells (B). **P 003C 0.01 and ***P 003C 0.001. [file 41065_2025_508_MOESM1_ESM.pdf]

Fig 4F

SUN-1

Control CA

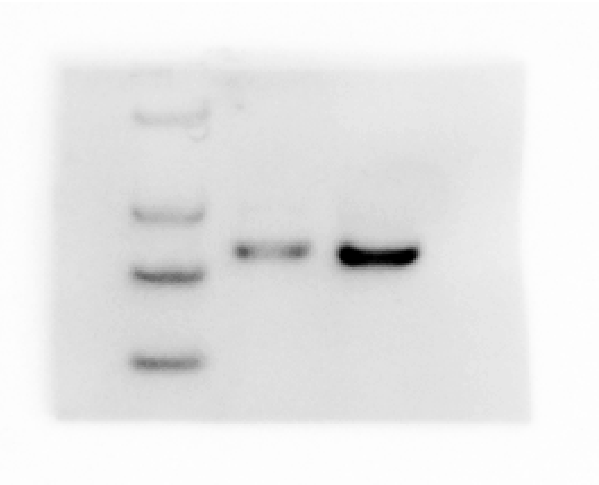

TP53
53kDa

Control CA

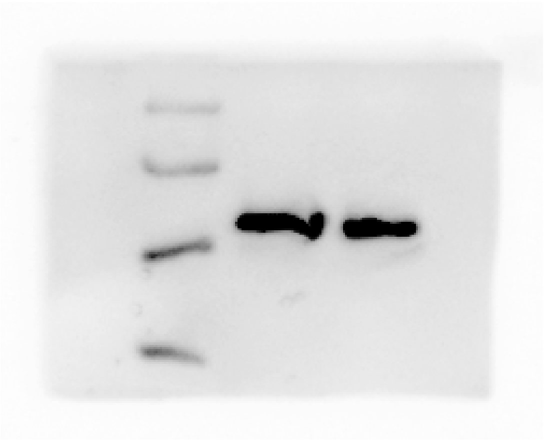

β-actin
43kDa

AGS

Control CA

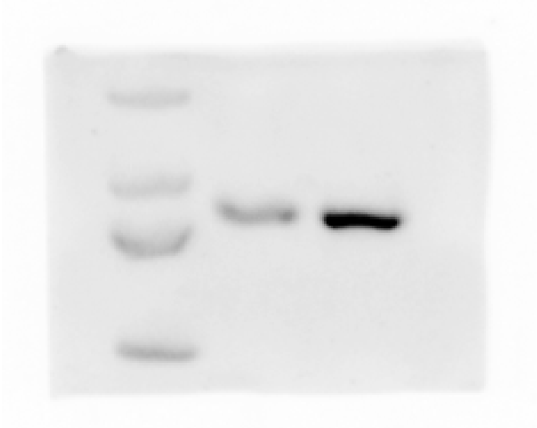

TP53
53kDa

Control CA

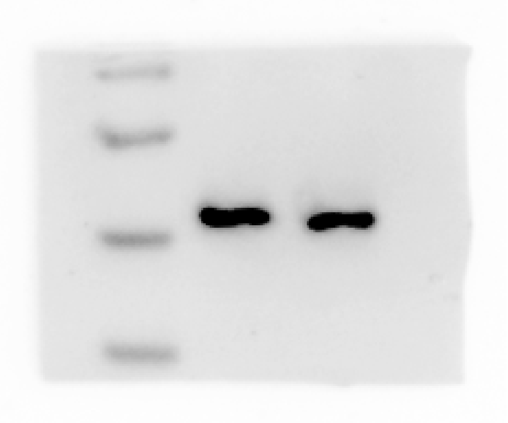

β-actin
43kDa

Fig 5A

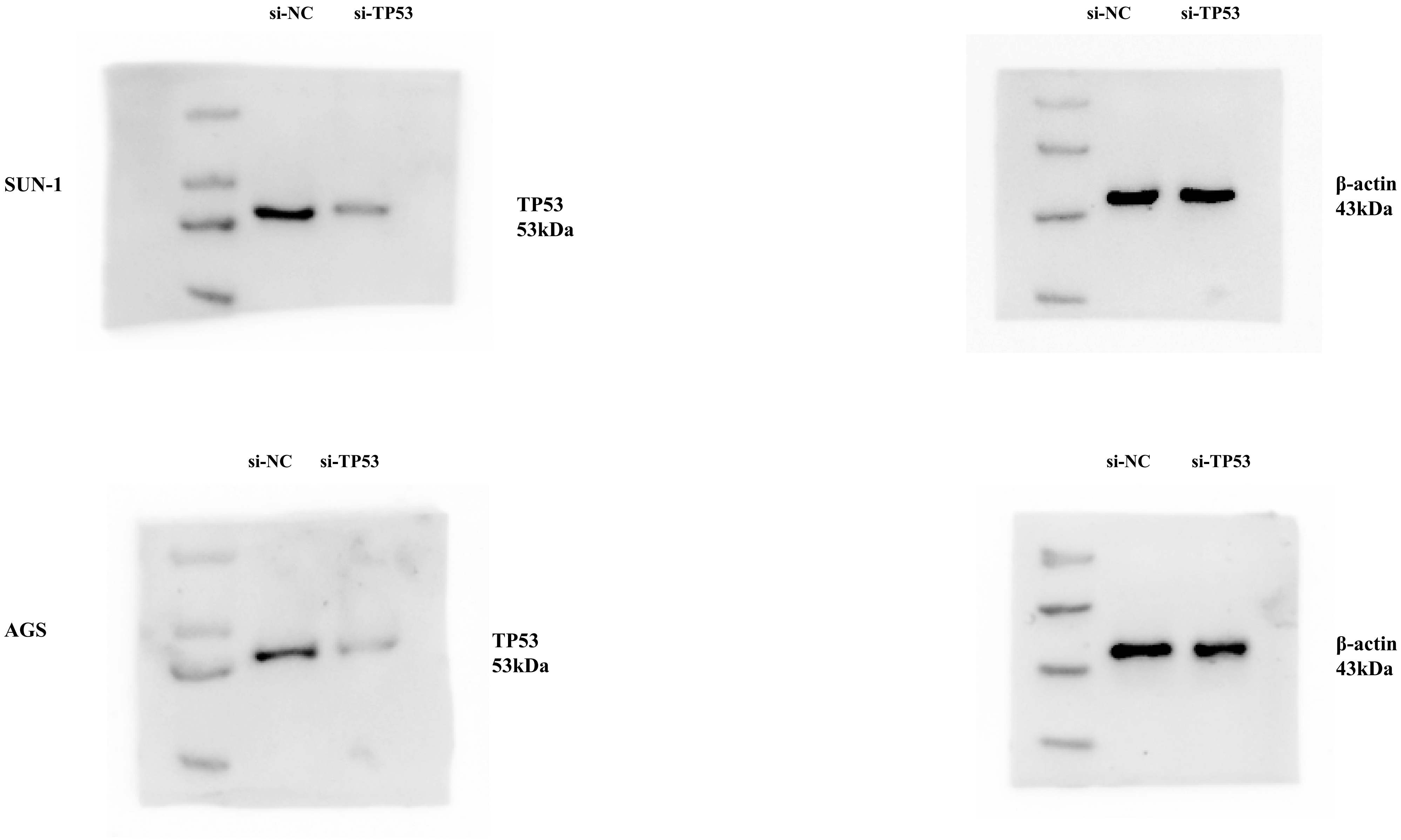
si-NC
si-TP53
SUN-1
TP53
53kDa
si-NC
si-TP53
β-actin
43kDa
si-NC
si-TP53
AGS
TP53
53kDa
si-NC
si-TP53
β-actin
43kDa

Fig 5B

SUN-1

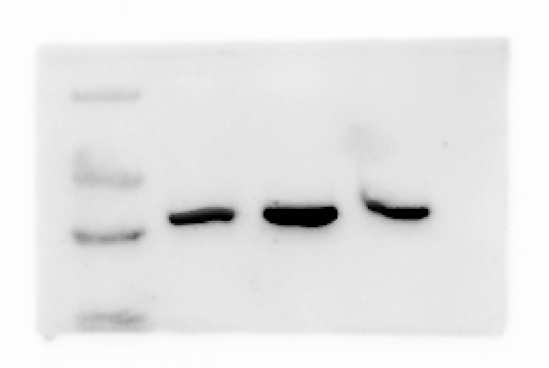

TP53
53kDa

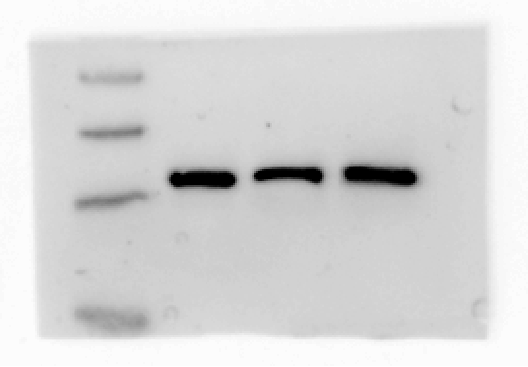

β-actin
43kDa

AGS

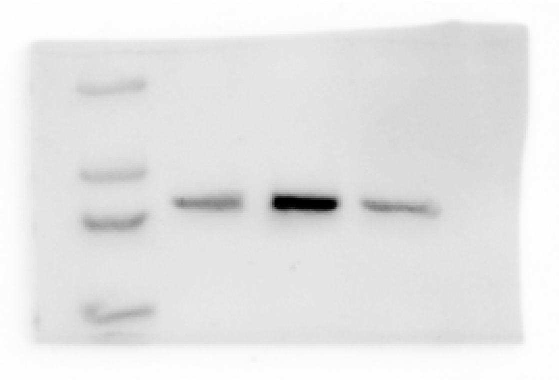

TP53
53kDa

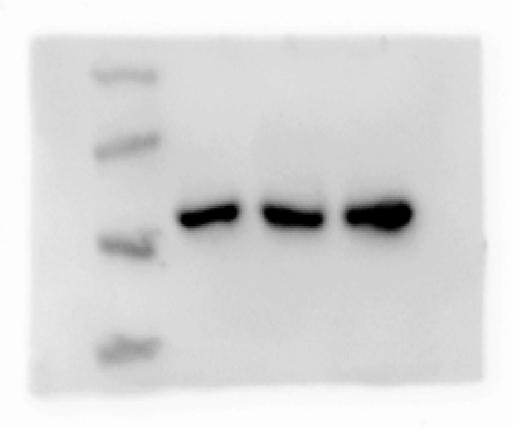

β-actin
43kDa

Fig 6A

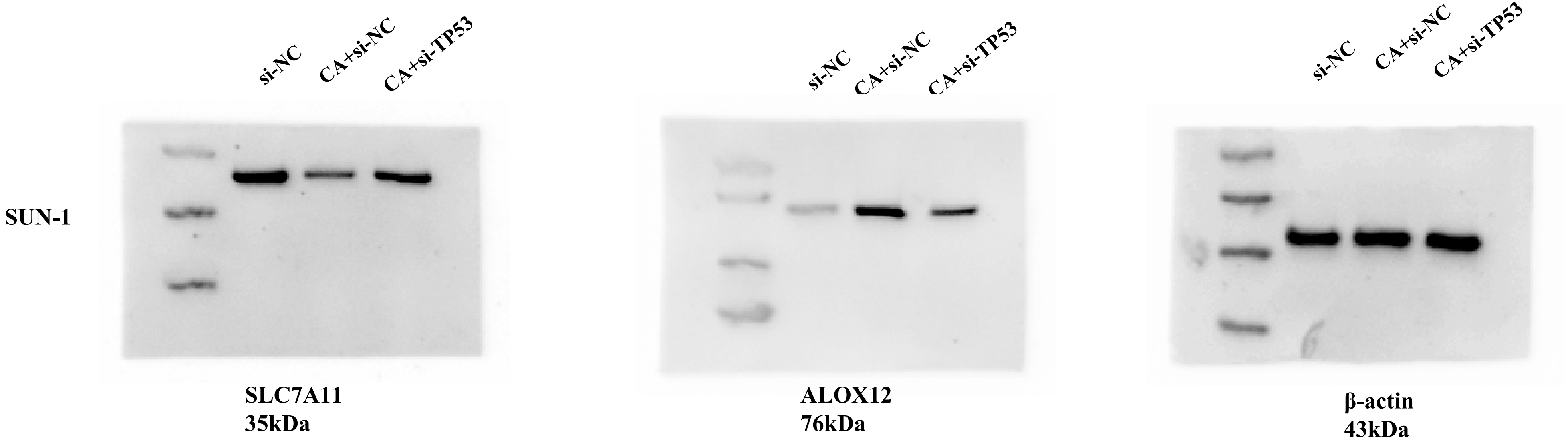
si-NC
CA+si-NC
CA+si-TP53
SUN-1
SLC7A11
35kDa
ALOX12
76kDa
β-actin
43kDa

Fig 6B

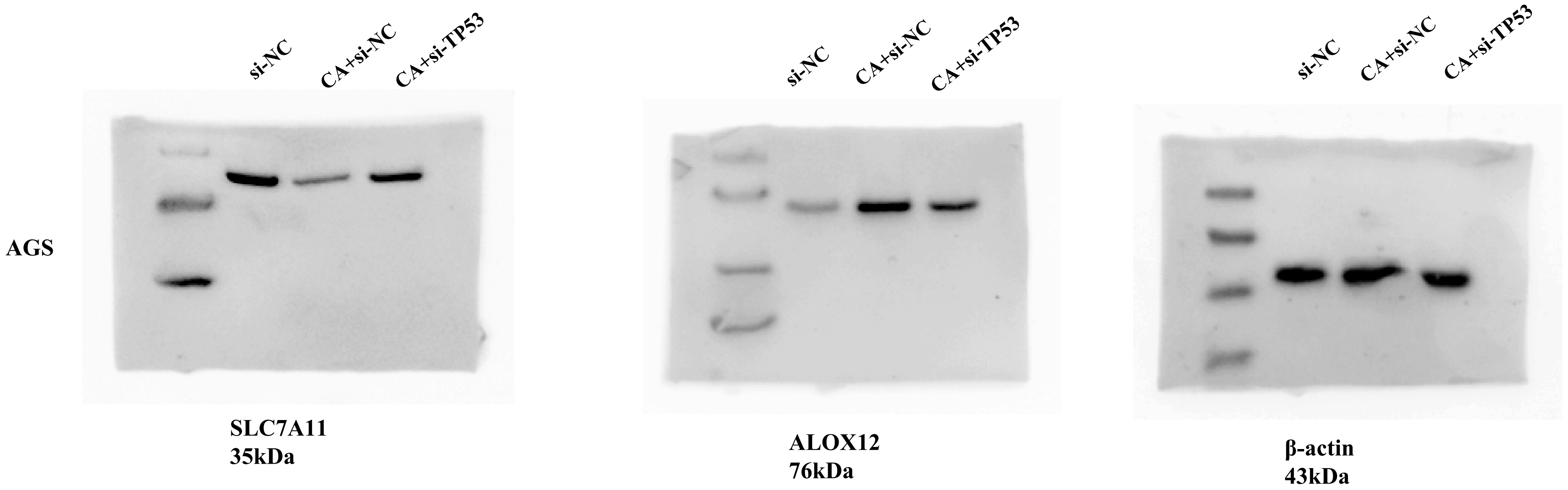
si-NC
CA+si-NC
CA+si-TP53
AGS
SLC7A11
35kDa
ALOX12
76kDa
β-actin
43kDa

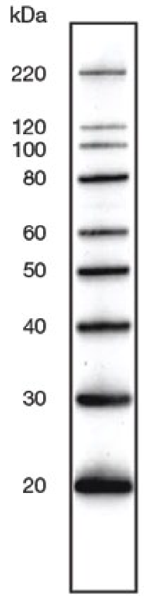

The samples derive from the same experiment and that gels/blots were processed in parallel.
